# *Candida albicans* adhesion to central venous catheters: Impact of blood plasma-driven germ tube formation and pathogen-derived adhesins

**DOI:** 10.1080/21505594.2020.1836902

**Published:** 2020-10-27

**Authors:** Philipp Jung, Clara E. Mischo, Gubesh Gunaratnam, Christian Spengler, Sören L. Becker, Bernhard Hube, Karin Jacobs, Markus Bischoff

**Affiliations:** a Institute for Medical Microbiology and Hygiene, Saarland University, Homburg, Germany; b Experimental Physics, Saarland University, Saarbrücken, Germany; c Department of Microbial Pathogenicity Mechanisms, Leibniz Institute for Natural Product Research and Infection Biology – Hans Knoell Institute Jena (HKI), Jena, Germany; d Institute of Microbiology, Friedrich Schiller University, Jena, Germany; e Max Planck School Matter to Life, Heidelberg, Jahnstr. 29, D-69120, Germany

**Keywords:** *Candida albicans*, microbial adhesion, germination, Als3, central venous catheters, single-cell force spectroscopy

## Abstract

*Candida albicans*-related bloodstream infections are often associated with infected central venous catheters (CVC) triggered by microbial adhesion and biofilm formation. We utilized single-cell force spectroscopy (SCFS) and flow chamber models to investigate the adhesion behavior of *C. albicans* yeast cells and germinated cells to naïve and human blood plasma (HBP)-coated CVC tubing. Germinated cells demonstrated up to 56.8-fold increased adhesion forces to CVC surfaces when compared to yeast cells. Coating of CVCs with HBP significantly increased the adhesion of 60-min germinated cells but not of yeast cells and 30-min germinated cells. Under flow conditions comparable to those in major human veins, germinated cells displayed a flow directional-orientated adhesion pattern to HBP-coated CVC material, suggesting the germ tip to serve as the major adhesive region. None of the above-reported phenotypes were observed with germinated cells of an *als3*Δ deletion mutant, which displayed similar adhesion forces to CVC surfaces as the isogenic yeast cells. Germinated cells of the *als3*Δ mutant also lacked a clear flow directional-orientated adhesion pattern on HBP-coated CVC material, indicating a central role for Als3 in the adhesion of germinated *C. albicans* cells to blood exposed CVC surfaces. In the common model of *C. albicans*, biofilm formation is thought to be mediated primarily by yeast cells, followed by surface-triggered the formation of hyphae. We suggest an extension of this model in which *C. albicans* germ tubes promote the initial adhesion to blood-exposed implanted medical devices via the germ tube-associated adhesion protein Als3.

## Introduction

*Candida albicans* is a common colonizer of mucosal surfaces of humans, and is present in 75% of the population [1,2]. While colonization by this fungus usually remains benign in healthy individuals, immunosuppressed patients are at increased risk of developing a variety of infections. For instance, oral candidiasis often affects the oropharynx and esophagus of patients infected by HIV. Other risk factors for oral candidiasis include advanced age or the wearing of dentures [3]. As a common infection of the female genital tract, vulvovaginal candidiasis affects both immunosuppressed and healthy women [4]. In contrast to superficial infections, systemic candidiasis and *Candida*-associated bloodstream infections are life-threatening conditions with mortality rates of up to 35%, even if adequate antifungal treatment is initiated [5–7].

*C. albicans* bloodstream infections are frequently related to infected medical devices such as central venous catheters (CVCs) [5]. CVCs are among the most commonly used intravascular devices in modern intensive care medicine. They are inserted into the internal jugular vein or, less frequently, into the subclavian vein or a femoral vein and serve different purposes, such as the administration of intravenous medications, parenteral nutrition, frequent blood draws or to quantify the central venous blood pressure [8]. However, in the case of catheter-related bloodstream infections (CRBSIs), CVCs are likewise a major reason of infection [9]. *Candida*-induced CRBSIs are usually driven by the formation of biofilms on CVCs, rendering the pathogen less susceptible to anti-infective therapy [10,11]. Mature *C. albicans* biofilms constantly release cells into the bloodstream, thereby “feeding” the infection. Given that the initial attachment of the pathogen to a catheter surface is a basic condition for biofilm formation, the pathogen’s ability to adhere to CVC surfaces can be considered a fundamental virulence feature.

*C. albicans* can form different morphotypes including the round to ovoid-shaped yeast phase (yeast cells) and the filamentous hyphal phase (hyphae) [2]. While the yeast phase is believed to serve as the major morphotype responsible for distribution and dissemination of *C. albicans* [12], the hyphal phase is considered to be the invasive infection form, able to actively penetrate epithelial cells, facilitate endocytosis, and to stabilize mature biofilms [2,13]. Different physiological triggers are known to induce the yeast-to-hyphal transition, such as an increasing pH, a temperature shift to 37°C, or changes in the availability of nutrients [14]. The formation of a polarized-growing germ tube on the yeast cell body marks the early steps of yeast to hyphal transition and will lead to the formation of a hypha after progressing elongation [15]. Furthermore, *C. albicans* is capable of performing mechanosensing: upon contact with a biotic/abiotic surface, yeast cells may induce the formation of germ tubes and hyphae [16]. Contact with human blood plasma (HBP) is another important transition trigger that promotes a morphology shift [17]. *C. albicans* is capable of interacting with a variety of proteins present in HBP and the extracellular matrix of host cells, such as fibronectin, fibrinogen, vitronectin, laminins, and different collagens [18–20]. The transition and interplay between the yeast and hyphal phases are important for the progression and regulation of infection processes [21].

However, the contribution of these two morphotypes to adhesion and biofilm formation is still not fully understood. Many studies described the yeast phase as the major morphotype being responsible for initial adhesion, followed by a surface-bound formation of germ tubes and the transition to the hyphal phase, triggered by e. g. mechanosensing or contact with blood [10,16,22,23]. However, other studies emphasize the importance of germ tubes/hyphae for the adhesion of *C. albicans* to endothelial- and epithelial cells [21,24–26]. All these studies agree that the hyphal phase is crucial for biofilm formation, whereas the contribution of the different morphotypes to the initial adhesion is less clear [27]. In accordance, hyphal phase-deficient mutants failed to form biofilms under *in vitro* conditions [28]. In this context, investigating germ tube/hypha-associated adhesins can provide important insights into the mechanisms of initial *C. albicans* adhesion. The agglutinine-like sequence glycoprotein 3 (Als3) is a multifunctional GPI-linked cell-surface factor, which is abundantly expressed on the germ tube [29–31], and described to promote adhesion to epithelial cells, endothelial cells, and extracellular matrix components, to facilitate cell invasion and iron uptake, and is expressed in maturating biofilms [17].

Here, we studied the impact of germination, HBP and Als3 on the initial adhesion of *C. albicans* yeast cells to commercially available CVC surfaces by single-cell force spectroscopy (SCFS). To learn more about the role of the germ tube on CVC adhesion under flow conditions comparable to those seen in major veins [32], we established a flow chamber model, using HBP-coated polyurethane used in CVC production as a substrate for adhesion.

## Material and methods

### Candida albicans *strain and cultivation conditions*

*C. albicans* (Robin) Berkhout strain DSM1386 (syn. ATCC 10231) was obtained from the German Collection of Microorganisms and Cell Cultures (DSMZ) and used for SCFS, flow chamber experiments, and the determination of yeast sizes and germ tube lengths. The *C. albicans* strain pair BWP17+Clp30 (referred to BWP17 in the following text) [33] and CAYF178U [34] were obtained from the stock collection of the Leibniz Institute for Natural Product Research and Infection Biology – Hans Knoell Institute Jena (HKI), Jena, Germany. Yeast cells were cultivated on trypticase soy agar (TSA) plates with 5% sheep blood or in yeast extract-peptone-dextrose (YPD) broth (pH 6.5) at 37°C. Media were purchased from Becton Dickinson GmbH, Heidelberg, Germany. Liquid cultures were aerated at 150 rpm in culture flasks with a flask to medium ratio of 10 to 1.

### Production of yeast cells and germinated cells

Yeast cells were grown in liquid YPD broth overnight and harvested by centrifugation at 5000 rpm for 5 min. The cell sediment was washed with sterile phosphate-buffered saline (PBS, pH 6.5) thrice and resuspended in PBS and adjusted to an optical density at 600 nm (OD_600_) of 1.5. The cell suspension was used either directly as a source for yeast cells in downstream applications, or to create germinated cells. To stimulate the yeast-to-hyphal transition, 200 µl aliquots of the OD_600_ 1.5 cell suspension were centrifuged as described above, and the cell sediments dissolved in equal volumes of human blood plasma (HBP; purchased from Sigma-Aldrich, Darmstadt, Germany). HBP cell suspensions were incubated at 37°C and 1000 rpm for 30 or 60 min to trigger germ tube formation, yielding 30-min germinated cells and 60-min germinated cells, respectively. Germinated cells were washed with PBS thrice to remove HBP prior to downstream applications.

### Determination of the radii of yeast cell bodies and germ tube lengths of germinated cells

Yeast cells were cultured and washed as described above, resuspended in prewarmed HBP or PBS, and subsequently incubated at 37°C and 1000 rpm for up to 300 min. At time points indicated, aliquots of the cell suspensions were removed and spotted on microscopic slides, covered with a coverslip, and monitored by bright field microscopy at 200x magnification using a DMI 4000 B optical microscope (Leica, Wetzlar, Germany). Ten pictures were taken per condition using a DFC 420 C camera with 15 fps (Leica, Wetzlar, Germany), and the radii/semi-axes of the mother cells and germ tubes were determined using the image analysis software ImageJ (version 1.8.0). For the determination of cell-surface areas, mean radii/semi-axes obtained for mother cells and germ tubes were used to calculate the surface areas by valuing mother cells as spheroids and germ tubes as round-shaped tubes with a hemisphere as tip. The calculated overall surface areas for germinated cells and yeast cells were subsequently used to calculate the factor for the increase in surface area seen with germinated cells in relation to yeast cells.

### Single-cell force spectroscopy (SCFS) on CVC surfaces

SCFS experiments were performed on a Flex-Bio Atomic Force Microscope in FluidFM® probe microscopy mode (Nanosurf GmbH, Liestal, Switzerland) and FluidFM micropipettes (Cytosurge AG, Glattbrug, Switzerland) with a spring constant of 0.3 N/m and an apex of 2 µm. To measure adhesion forces, *C. albicans* yeast and germinated cell dilutions (~10^5^ cells/ml) were placed in polystyrene petri dishes. Single *C. albicans* cells were brought into contact with the FluidFM micropipette, and immobilized on the probe apex by negative pressure of −600 mbar to create cell-functionalized AFM probes. The cells were grabbed on the apical top of the cell body. When germinated cells were probed a vertical germ tube-orientation relative to the AFM cantilever was ensured by optical microscopy. All SCFS measurements were conducted in PBS. For further information on FluidFM-based SCFS with *C. albicans* cells, the reader is referred to [35].

Three commercially available polyurethane (PU)-based CVC types from different manufacturers (B. Braun, Melsungen, Germany [I], Vygon, Aachen, Germany [II], and Arrow®, Teleflex Medical GmbH, Fellbach, Germany [III]), used in daily clinical practice at the Saarland University Medical Center, Homburg, Germany, were probed. An approximately 5 mm long piece of the lower end of the CVC tube was radially cut to create a halved CVC tube fragment, which was subsequently fixed in the middle of a polystyrene petri dish using adhesive tape. The fixed CVC piece was covered with a droplet of HBP for 30 min at 37°C and washed thrice with PBS to achieve a coating with plasma factors. SCFS was conducted on the apical top of the halved CVC tube with a force trigger of 6 nN, a ramp size of 5 µm, and variable surface delay times ranging from 0 s to 1 s. Per fungal cell, 16 individual force–distance curves were recorded on the CVC surface with a lateral distance between two force–distance curves set to 1 µm. After exchanging the fungal cell, a new spot on the catheter was probed. The values summarized in Figures 2, 3 and 5 are displayed as adhesion force and rupture length averages per cell. The number of investigated cells is given in the figure legend of each force spectroscopy experiment. Force–distance curve analyses were conducted with the SPIP software version 6.6.2 (Image Metrology, Hørsholm, Denmark).

### Flow chamber experiments on CVC material

*C. albicans* yeast cells, 30-min germinated cells, and 60-min germinated cells were channeled through bottomless ibidi Sticky-Slide chambers, operated by the ibidi Pump System Quad (ibidi GmbH, Gräfeling, Germany) with 4 dyn/cm^2^ and 1.6 mm perfusion sets. The bottomless chambers were mounted on Tecoflex® EG 85A (Velox GmbH; Hamburg, Germany), which is thermoplastic polyurethane (PU) material used to manufacture PU-based CVCs. To coat the surface with plasma factors, HBP was pipetted into the flow chamber sandwiches, incubated for 30 min, and flushed with PBS (4 dyn/cm^2^) for 2 min. Cell adhesion kinetics and germ tube-direction patterns were observed with the microscopic setup described above. A minimum of nine pictures were analyzed per condition. To distinguish between adherent cells and cells in flow, the optical focus was shifted and the camera settings were set to an exposure time of 200 ms and 2.6-fold digital gain, creating photographic triplets representing cell movement in flow direction.

### Statistical analyses

GraphPad Prism (Version 6, GraphPad, San Diego, USA) was used to test for statistically significant differences. *P* values <0.05 were considered significant. The statistical tests used are described in the relevant figure legends.

## Results and discussion

### C. albicans *germ tube formation in HBP*

**Figure 1.**
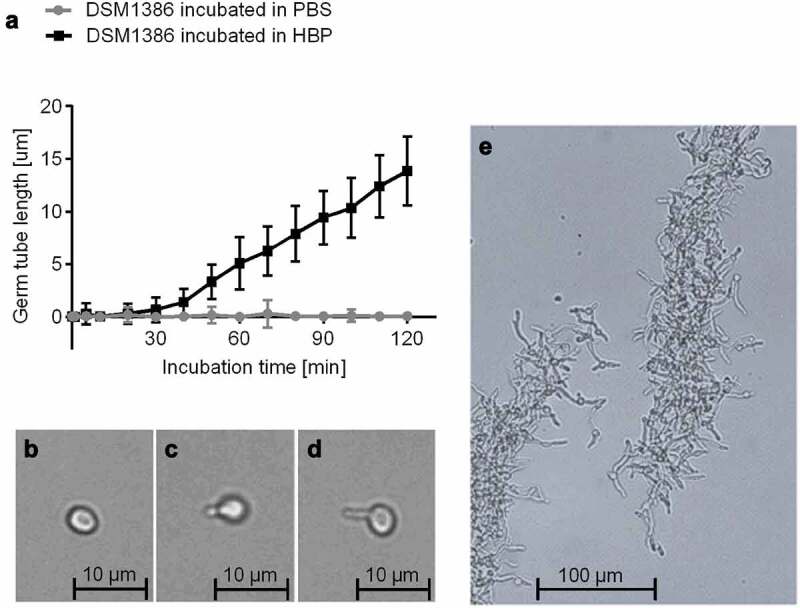
**Impact of the HBP incubation time on *C. albicans* germination. A**: *C. albicans* WT DSM1386 yeast-to-hyphal transition and germ tube formation in human blood plasma (HBP) in vitro at 37°C and 1000 rpm. The mean germ tube lengths of *C. albicans* cells incubated in PBS or HBP were measured. Thirty *C. albicans* cells were observed per time point and condition **B-D**: Representative micrographs of a yeast cell incubated in PBS for 60 min (b), a 30-min germinated cell (c), and a 60-min germinated cell (d). **E**: Hyphae formation after 180 min incubation in HBP

Different physiological triggers are known to induce the transition from yeast to hyphae [17]: The presence of HBP and a temperature of 37°C, conditions found in the bloodstream of humans, are described as especially strong triggers [14]. In order to determine the germination kinetics of wild type (WT) strain DSM1386 in HBP, yeast cells were cultured overnight in YPD broth, transferred to PBS or HBP, and subsequently incubated at 37°C and 1000 rpm for up to 300 min (Figure 1). While the PBS-incubated cells retained the yeast morphotype (Figure 1a,b), cells incubated in HBP underwent a yeast-to-hyphal transition by forming visible germ tubes with length of 0.9 ± 0.6 µm after 30 min of incubation (Figure 1a,c). After 60 min, these germ tubes reached a length of 5.1 ± 2.5 µm and further elongated almost linearly along with time (Figure 1a,d). The *C. albicans* cells induced as described are referred to as 30-min germinated cells and 60-min germinated cells in the following sections. *C. albicans* yeast cells incubated in HBP for 180 min and longer demonstrated the formation of biofilm-resembling floating aggregates with interlaced, stabilizing hyphae (Figure 1e).

### *Impact of the germ tube on the adhesion of* C. albicans *yeast cells to naïve CVC surfaces from different manufacturers*

**Figure 2.**
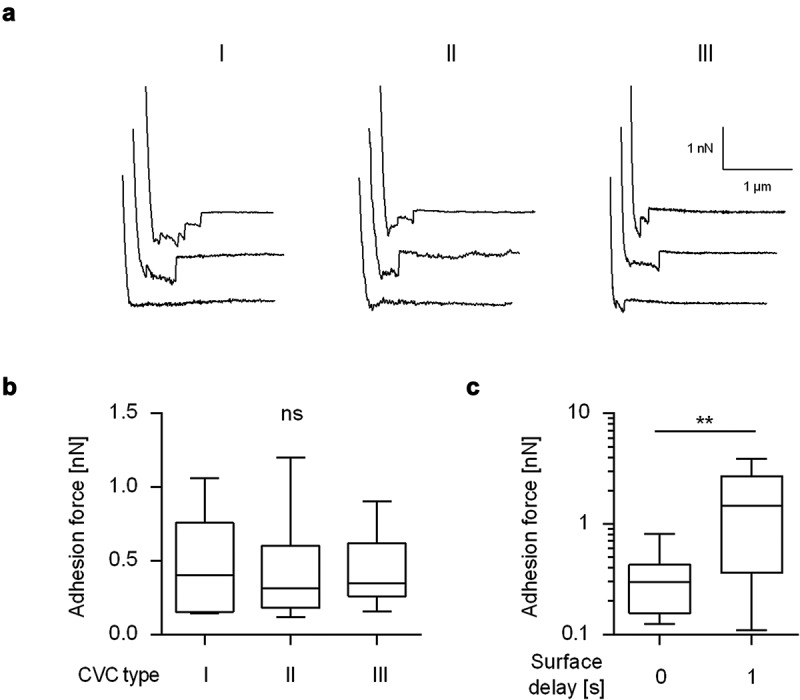
**Maximum adhesion forces of *C. albicans* yeast cells to naïve CVC surfaces from different manufactures**. Individual yeast cells incubated for 30 min in PBS were immobilized on FluidFM micropipettes and used for SCFS on naïve CVC surfaces from three different manufacturers (I to III). **A**: Representative retraction curves collected from cells that were probed with CVC surfaces from manufacturers I to III without any surface delay (0 s). **B**: Mean adhesion forces obtained from force/distance curves recorded on CVC surfaces from manufacturers I to III with a surface delay time of 0 s. Data are shown as a box and whisker plot (min-to-max) representing the mean values of 10 individual cells per CVC type. ns, not significant (Kruskal-Wallis test with Dunn’s post hoc test). **C**: Influence of the surface delay time on the adhesion forces. Yeast cells were probed with the CVC surface with different contact times (0 s and 1 s). Data are shown as a box and whisker plot (min-to-max) representing the mean values of 10 individual cells per time point and condition. ns, not significant; **, *P*< 0.01 (Mann-Whiney *U* test)

Blocking germ tube formation of *C. albicans* by farnesol or *Saccharomyces boulardii* cell extracts was shown to reduce the adhesion of this yeast to polystyrene [36], and some of the adhesins of this pathogen, such as Als3, are expressed predominantly in germinated cells [29]. However, the exact role of *C. albicans* germ tubes on the adhesion of this pathogen to CVC surfaces has, to the best of our knowledge, not been experimentally investigated yet. In order to fill this gap, FluidFM-based SCFS was used here to study the adhesion behavior of WT DSM1386 yeast cells and germinated cells on CVCs as a commonly used implant material. In a first step, SCFS was applied to measure the adhesion forces of yeast cells on three commercially available polyurethane (PU)-based CVC types obtained from different manufactures (Figure 2). Yeast cells of strain DSM1386 displayed on all tested naïve CVC tubing comparable retraction force profiles (Figure 2a), and maximum adhesion forces that were in a range of 0.12 nN to 1.2 nN (Figure 2b) were observed. Most of the retraction force profiles obtained with yeast cells on the naïve CVC surfaces exhibited longer extensions (up to 1.23 µm; mean 0.52 ± 0.34 µm, n = 12 individual cells) and a few smaller peaks, indicating the unbinding and unfolding of high molecular weight *C. albicans* multidomain cell-surface components [37]. Variations of the surface delay time – the timespan the cell is kept in contact with the substratum before the probe is removed from it to record the retraction force profile – from 0 to 1 s increased the adhesion force between the yeast cell and the naïve CVC surface by a factor of about 4 (Figure 2c), which is in line with earlier findings with *C. albicans* strain SC5314 on dodecyl phosphate-coated glass wafers [35]. These findings suggest that the prolongation of the contact time allowed for a stronger adhesion of the yeast cell to the implant material.

**Figure 3.**
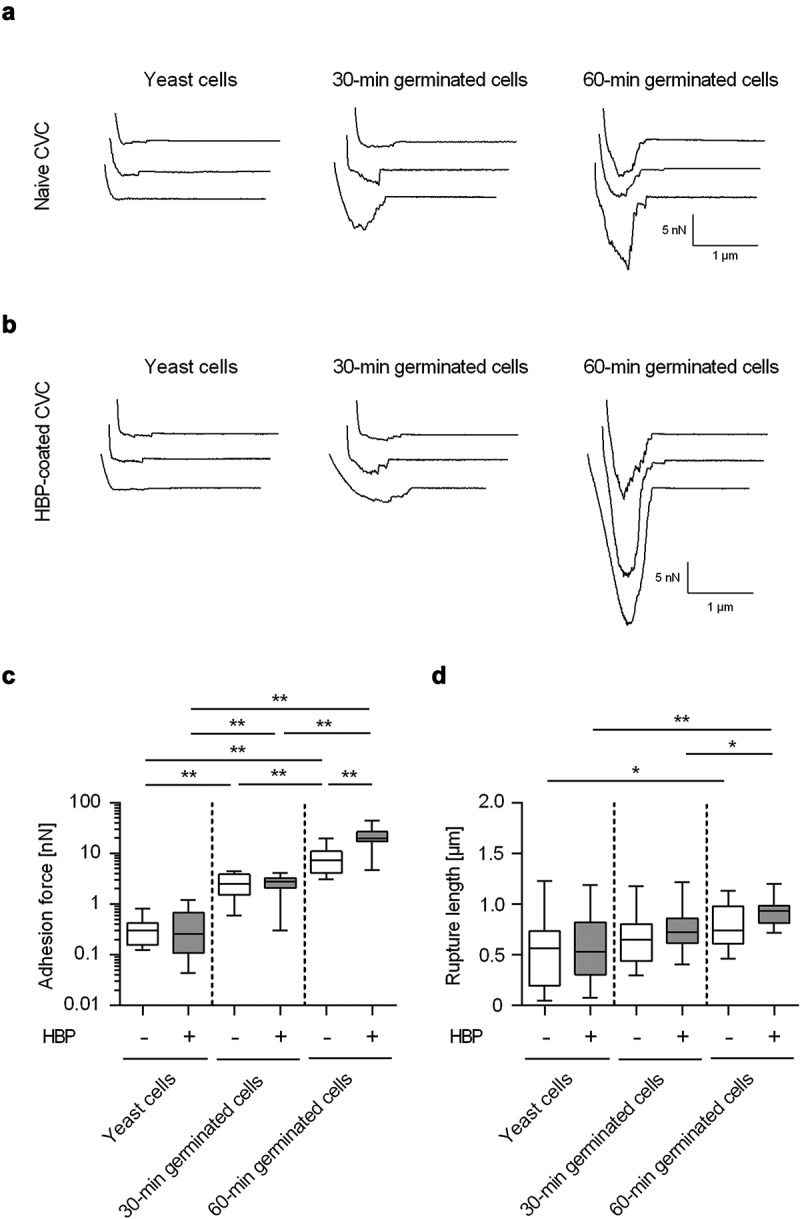
**Impact of germination on the adhesion forces of *C. albicans* cells to naïve and HBP-coated CVC surfaces**. Cells were either incubated in PBS (yeast cells) or HBP (30-min and 60-min germinated cells). Individual cells were immobilized on FluidFM micropipettes and used for SCFS on naïve or HBP-coated tubing of CVC type I (surface delay time 0 s). **A, B**: Representative retraction curves obtained with the cell types indicated on untreated (a) or HBP-coated (b) CVC surfaces. **C, D**: Maximum adhesion forces (c) and rupture lengths (d) on naïve (white boxes) or HBP-coated (gray boxes) CVC surfaces. Data are shown as box and whisker plots (min-to-max) representing the mean values obtained from ≥10 individual cells per condition. *, *P*< 0.05; **, *P*< 0.01 (Mann Whitney *U* test)

In a next step, SCFS was used to determine the adhesion behavior of germinated cells on naïve CVC surfaces (Figure 3a-d). Here, force spectroscopy with *C. albicans* WT strain DSM1386 revealed up to 6.6- and 56.8-fold increased adhesion forces for 30-min germinated cells and 60-min germinated cells, respectively (Figure 3c). Notably, germ tube formation still augmented the adhesion forces of the germinated cells by factors of 5.6 and 36.9-fold, when accounting for the increased surface areas caused by germination (calculated from the microscopically determined sizes of the cell body and germ tube; factors 1.18 and 1.54, when compared to the surface areas of yeast cells). However, when the impact of germination on the maximum rupture length between the fungal cell and the naïve CVC surface was monitored, more minor changes were observed (Figure 3d). Only 60-min germinated cells displayed significantly enhanced rupture lengths when compared to yeast cells, while the rupture lengths of 30-min germinated cells remained in the same range as the ones seen with yeast cells on the naïve CVC surface (Table 1). These observations suggest that germ tubes formed by 60-min germinated cells may express adhesins on the cell-surface capable of inducing a qualitative change in adhesion between the fungal cell and the CVC tubing.

**Table 1.** Adhesion forces and rupture lengths of yeast cells, 30-min and 60-min germinated cells on untreated and HBP coated CVC surfaces

Table 1	Adhesion forces [nN]			Rupture lengths [nm]		
	Surface delay [s]		*P*^1^	Surface delay [s]		*P*^1^
	0	1		0	1	
Untreated CVC surfaces^2^						
Yeast cells	0.33 ± 0.2	1.56 ± 1.2	**	519 ± 338	607 ± 299	ns
30-min germinated cells	2.61 ± 1.3	4.78 ± 2.8	*	649 ± 264	628 ± 216	ns
60-min germinated cells	7.86 ± 4.4	16.55 ± 9.6	**	766 ± 203	715 ± 145	ns
HBP-coated CVC surfaces^2^						
Yeast cells	0.39 ± 0.3	1.31 ± 0.7	**	586 ± 329	576 ± 190	ns
30-min germinated cells	2.59 ± 1.0	4.78 ± 2.2	**	752 ± 195	782 ± 128	ns
60-min germinated cells	22.17 ± 9.9	36.2 ± 18.3	*	911 ± 127	907 ± 151	ns

^1^*, *P*< 0.05; **, *P*< 0.01; Mann-Whitney *U* test

^2^Mean values and SD obtained from 10–20 individual cells per condition and cell type

### *Impact of HBP-coating of CVCs on the adhesion properties of* C. albicans

Since CVC surface regions are rapidly covered with blood factors following insertion into a central vein [38,39], we aimed to test the impact of an HBP-coating of the CVC surface on the adhesion forces of *C. albicans* yeast cells and germinated cells. To our surprise, HBP-coating of the CVC surface did not markedly affect the adhesive potentials of yeast cells and 30-min germinated cells (Figure 3c-d). However, 60-min germinated cells of WT DSM1386 demonstrated significantly increased adhesion forces to HBP-coated CVC surfaces when compared to uncoated surfaces (Figure 3c). Increasing the surface delay of the SCFS probes from 0 s to 1 s enhanced the maximum adhesion forces observed for yeast cells on HBP-coated CVC tubes. This effect was also observed for both types of the germinated cells, which all displayed enhanced maximum adhesion forces on HBP-coated CVC tubes with the higher contact time tested (Table 1). The enhanced contact time did not markedly affect the maximum rupture lengths recorded for all three *C. albicans* cell types on naïve and HBP-coated CVC tubes (Table 1), but 60-min germinated DSM1386 cells that were brought into contact with the CVC surface for 1 s displayed increased rupture lengths on HBP-coated CVC surfaces when compared to uncoated surfaces (*P*= 0.007, Mann-Whitney *U* test).

Taken together, this series of experiments yielded several new insights: (i) Germinated *C. albicans* cells display an enhanced adhesion capacity to commercially available PU-based CVCs compared to yeast cells. We believe that this finding adds an important new aspect to the understanding of *C. albicans* biofilm formation on implanted medical devices, in addition to the already well-described mechanism of adhering yeast cells, followed by secondary, surface-triggered formation of hyphae on the artificial surface [16,40], in which a specific role for the germ tube in *C. albicans* adhesion to implant material was not considered yet; (ii) Bacterial AFM studies suggest that microbial cell adhesion to an abiotic surface is primarily driven by charge and hydrophobic/-philic interactions, and that proteinaceous factors on the bacterial cell-surface play a major role in this process [41–43]. An important role for surface proteins in cell adhesion to abiotic surfaces was also reported for the closely related species *Candida glabrata* [37,44]. The retraction curves obtained with yeast cells of WT DSM1386 indicate that this holds also true for *C. albicans* and PU-based CVC surfaces (Figure 2a); (iii) However, coating the surface of such an implanted device with body fluid components is likely to add another aspect to the investigated adhesion process, which is host factor–pathogen interaction. HBP incubation of the CVC leads to the deposition of blood factors, mostly proteins, such as albumin, fibrinogen, fibronectin, or vitronectin that are rapidly immobilized on the CVC surface upon insertion into the vessel system [39]. It can be assumed that these immobilized host factors can be bound by specific *C. albicans* adhesins such as the agglutinin-like sequence protein family (Als1-7, 9), the hyphal wall protein 1 (Hwp1), or other GPI-linked proteins (e.g. Eap1, Iff4, Ecm33) [17,19]. The enhanced adhesion forces and rupture lengths seen with 60-min germinated cells on HBP-coated CVCs, when compared to uncoated CVCs, are in line with this assumption and suggest that specific binding of immobilized plasma proteins by *C. albicans* adhesins contributes to this phenomenon. These *C. albicans* adhesins might not be present – or expressed to a smaller extent – on yeast cells and 30-min germinated cells, which might explain why no clear differences in adhesion forces and rupture lengths between HBP-coated and uncoated CVC surfaces were observed for the latter two cell types (Figure 3). (iv) Significant differences in the adhesion forces were observed for all three cell types, when contact times between cells and the CVC surfaces were varied between 0 s and 1 s (Figure 3 and Table 1). Thus, *C. albicans* seems to be able to augment its adhesive strength toward this commonly used implant material along with contact time, which probably facilitates the residence time of the fungal cell on the CVC surface under high flow rates and shear stress present in larger blood vessels.

### *Adhesion of* C. albicans *on HBP-coated CVC-like material under flow*

**Figure 4.**
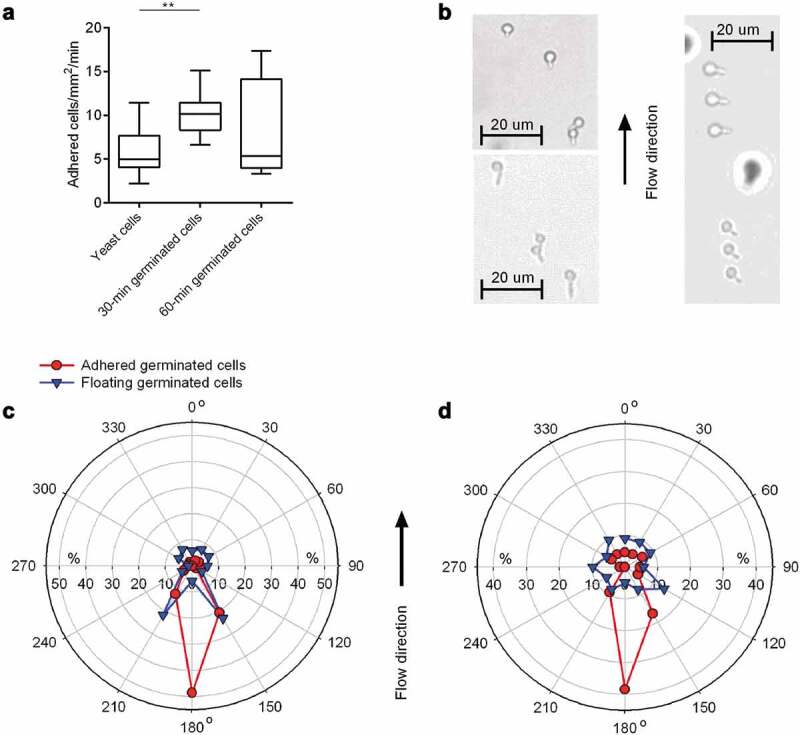
**Impact of germination on floating and adhesion behaviors of *C. albicans* cells**. 30-min and 60-min germinated cells were channeled through Tecoflex® EG 85A-functionalized and HBP-coated ibidi Sticky-Slide chambers with a flow rate of 4 dyn/cm^2^. **A**: Adhesion kinetics of yeast cells, 30-min and 60-min germinated cells on HBP-coated Tecoflex® EG 85A after 30 min in flow. Data are shown as box and whisker plots (min-to-max) representing counts of adherent cells/mm^2^/min. **, *P*< 0.01 (Mann Whitney *U* test). **B**: Representative images of 30-min germinated cells (top left image), 60-min germinated cells (bottom left image) and floating 60-min germinated cells as triplets (right image; sessile cells are out of optical focus and surrounded by a halo). **C, D**: Germ tube angles of floating (blue symbols) and attached (red symbols) germinated cells of WT DSM1386 relative to the flow direction. Percentages of angle frequencies are displayed as polar plots for 30-min germinated cells (c), and 60-min-germinated cells (d). For every condition, a minimum of 100 and 70 germ tube angles were measured for adherent and floating cells, respectively

When *C. albicans* gets first into contact with implanted CVC tubing via hematogenous dissemination, the fungal cells have to adhere to the medical device under shear stress elicited by blood circulation. Earlier work demonstrated that, despite of the presence of hypha-repressing neutrophils, a large portion of *C. albicans* cells forms germ tubes in human blood [45]. It was also shown that *C. albicans* yeast cells and hyphal forms can bind to an endothelial cell monolayer under flow conditions that mimic the conditions found within blood vessels [46,47]. However, contradictory results were published regarding the adherence frequencies of yeast cells and hyphal forms under conditions of flow. While Grubb and colleagues [46] reported that yeast cells bound to the endothelial cell monolayer under flow in significantly higher numbers than hyphal forms, the exact opposite was observed by Wilson and Hube [47]. To test whether and how germ tube formation might influence the binding capacity of WT DSM1386 cells to CVC tubing under flow, we set up a flow chamber model, in which yeast cells or germinated cells were channeled through a flow chamber (flow rate: 4 dyn/cm^2^), and the floating- and adhesion behavior of the cells to the HBP-coated PU material Tecoflex® EG 85A was monitored by light microscopy (Figure 4). When yeast cell solutions were channeled through the flow chamber for 30 min, about 5 cells/mm^2^/min bound to the HBP-coated PU material (Figure 4a). However, when an equal amount of 30-min germinated cells were channeled through this device, about twice as much cells adhered to the HBP-coated PU material as has been observed for yeast cells. Surprisingly, for 60-min germinated cells, no clear difference in the adhesion frequency could be observed in this system when compared to yeast cells, supporting the findings made by Wilson and Hube [47] showing that germinated cells producing an early-stage germ tube are better suited to adhere to an endothelial cell layer under flow than yeast cells and germinated cells displaying a more advanced hyphal morphology.

The formation of a germ tube generates a distinct geometric change that might have a substantial impact on the mechanism of adhesion. Under flow conditions, germ tubes might influence the floating behavior of *C. albicans* and should be considered as well. In order to test this hypothesis, we determined the orientation of germ tubes of floating germinated cells in relation to the flow direction. Following the idea raised by Wilson and Hube [47] that the orientation of the germ tube of adherent cells in relation to the flow direction provides further insights into which part of the germinated cells are likely to represent the major focus of adhesion, we additionally determined the orientation of germ tubes of adherent germinated cells in relation to the direction of flow (Figure 4b-d). Wilson and Hube [47] reasoned that if the tip of the germ tube is the major focus of adhesion, germ tubes should adhere with the mother cell aligned in flow direction; conversely, if the mother cell represents the major focus of adhesion, germ tubes should adhere with the mother cell directed against the direction of flow; finally, if adhesion is neither specifically mediated by the mother cell nor the germ tube tip, germ tubes of adherent cells should be oriented in a random fashion. We observed that germ tubes of floating 30-min germinated cells showed a mild tendency to flow in the system with the germ tube being orientated in a + 30° and −30° angle against the direction of flow (i.e. 150° and 210°; Figure 4c), while germ tubes of floating 60-min germinated cells displayed an almost random orientation of the germ tubes (Figure 4d), suggesting that germinating cells producing an early-stage germ tube tend to flow in this system with a preferred floating orientation, while this is not the case with germinated cells displaying a more advanced hyphal morphology.

When the germ tube orientation of adhered germinated cells was determined, a completely different picture emerged. Germ tubes of germinated cells that adsorbed to the HBP-coated substrate demonstrated a clear flow directional orientation (Figure 4c,d). The vast majority of the germ tubes of adherent 30-min and 60-min germinated cells tethered to the surface with the germ tube pointing against the direction of flow (i.e. with an angle of 150°-210°). This flow directional-orientated adhesion pattern suggests that germ tubes fulfill an anchoring function [47], and promote adhesion of the germinated cell upon contact with the HBP-coated CVC material. This assumption implies that the germ tube and especially its apical tip harbor structures facilitating adhesion, which are less pronounced or not present on the corresponding mother cell. Putative factors are the adhesins Als1, Als3, and Hwp1, which are predominantly expressed on the germ tubes of germinated *C. albicans* cells but not or only rarely on mother cells [29,31,48,49].

### *Impact of Als3 on the adhesion properties of* C. albicans *to HBP-coated CVC tubing and HBP-coated CVC-like material under flow.*

**Figure 5.**
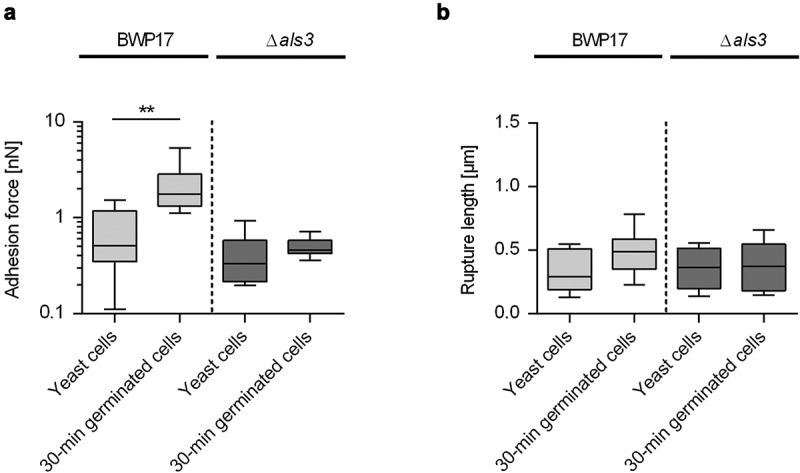
**Influence of Als3 on *C. albicans* adhesion to HBP-coated CVC surfaces**. Cells were incubated in HBP for 30 min or kept in PBS. Individual cells were immobilized on FluidFM micropipettes and used for SCFS on HBP-coated tubing of CVC type I (surface delay time 0 s). **A, B**: Maximum adhesion forces (a) and rupture lengths (b) of *C. albicans* BWP17 and its *als3*Δ/*als3*Δ mutant CAYF178U. Data are shown as box and whisker plots (min-to-max) representing the mean values obtained from 9 individual cells per condition (**, *P*< 0.01 Mann-Whitney *U* test)

Earlier work demonstrated that the transcription of the *ALS3* gene is strongly upregulated in germinating *C. albicans* cells [50], that the protein Als3 is predominantly found on the germ tubes of germinated cells [29–31,50], that Als3 displays different adhesion properties on the germ tube than on the mother cell [29], and that Als3 contributes to the enhanced cell-surface hydrophobicity reported for germ tubes [29]. To elucidate whether and how Als3 contributes to the enhanced adhesion phenotypes seen with germinated WT DSM1386 cells on CVC tubing and the flow directional-orientated adhesion pattern, we made use of an isogenic *C. albicans* WT/*als3*Δ strain pair, the SC5314 derivative BWP17 and its *als3*Δ/*als3*Δ mutant CAYF178U [33,34]. Upon incubation with HBP for 30 min, germinated cells of BWP17 and CAYF178U produced comparable germ tube length (0.9 ± 0.3 µm and 0.8 ± 0.3 µm, respectively), which were in the range of the germ tube lengths produced by 30-min germinated cells of WT DSM1386 (Figure 1a). When BWP17 yeast cells and 30-min germinated cells were probed by SCFS on HPB-coated CVC tubing, a significant increase in maximum adhesion forces was observed for the 30-min germinated cells (Figure 5a), confirming our findings made with WT strain DSM1386 (Figure 3c). Notably, when yeast cells and 30-min germinated cells of the *als3*Δ/*als3*Δ mutant were probed under these conditions, no clear differences in maximum adhesion forces were observed between both cell types (0.50 ± 0.11 nN and 0.40 ± 0.25 nN for the 30-min germinated cells and the yeast cells, respectively; *P*= 0.1359 [Mann-Whitney *U* test]). When rupture lengths were compared (Figure 5b), no clear differences between yeast cells and 30-min germinated cells were observed, in line with our findings made with WT DSM1386 cells (Figure 3d). These findings suggest that Als3 is a major factor contributing to the enhanced adhesion behavior of germinated cells to PU-based CVC tubing, which is in line with earlier findings demonstrating an important role for Als3 in the adhesion process of germinated *C. albicans* cells to different epithelial and endothelial cell types [50]. Additionally, a more minor role for the increase in surface area associated with germ tube formation can be assumed for the adhesion behavior of early stage-germinating *C. albicans* cells to CVC tubing, given that yeast cells and 30-min germinated cells of the *als3*Δ/*als3*Δ mutant adhered with comparable forces to the HBP-coated medical device.

**Figure 6.**
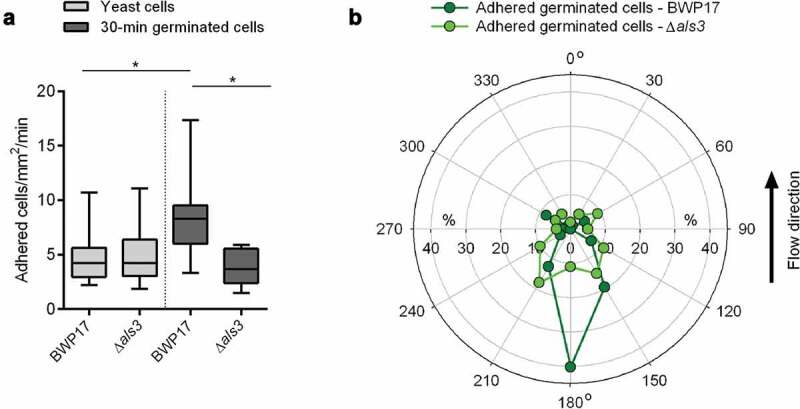
**Impact of Als3 on adhesion and germ tube orientation of C. *albicans* cells adhered in flow**. 30-min germinated cells were channeled through a flow chamber with a flow rate of 4 dyn/cm^2^. **A**: Adhesion kinetics of *C. albicans* BWP17 and its *als3*Δ/*als3*Δ mutant (*als3*Δ) after 30 min. Data are shown as box and whisker plots (min-to-max) representing counts of adherent cells/mm^2^/min. *, *P*< 0.05 (Mann Whitney *U* test). **B**: Germ tube angles of attached 30-min germinated cells of BWP17 (dark green symbols) and its *als3*Δ*/als3*Δ mutant (light green symbols) relative to the flow direction. Percentages of angle frequencies are displayed as polar plot, based on a minimum of 90 germ tube angles per condition

Next, 30-min germinated cells and yeast cells of the strain pair BWP17/CAYF178U were channeled through the flow chamber system as described above, and the adhesion frequencies and orientation of germ tubes of adherent cells tethered to the HBP-coated PU material were determined (Figure 6a,b). In line with our observations made with WT DSM1386 cells, we detected a significant increase in the number of germinated cells of strain BWP17 attaching to the surface, when compared to the corresponding yeast cells (Figure 6a). However, this effect was not seen with 30-min germinated cells of the *als3*Δ mutant, which adhered with a similar frequency to the HBP-coated PU material as yeast cells of this mutant. Notably, 30-min germinated *als3*Δ mutant cells displayed a similar adhesion frequency to the HBP-coated PU surface as yeast cells of the parental strain (Figure 6a), suggesting that Als3 expressed on the germ tubes of germinated *C. albicans* cells is primarily responsible for the increase in adhesion under flow.

When the orientation of the germ tubes of surface-attached germinated BWP17 cells was determined, a similar flow directional orientation as for adherent germinated DSM1386 WT cells was observed (Figure 4c), with nearly half of the germ tubes of adherent cells orientating almost exactly against the direction of flow (Figure 6b), supporting our hypotheses that germ tubes display an anchoring function and promote adhesion upon contact with HBP-coated abiotic materials, and that the apical tip of the germ tube is likely to harbor structures facilitating this adhesion. Germinated CAYF178U cells that adhered to the HBP-coated PU material under flow, on the other hand, displayed no such flow directional orientation and tethered with almost random germ tube orientation to the surface (Figure 6b), suggesting that Als3 expressed on the germ tube tip is a major factor for this binding characteristic.

## Conclusion

AFM-based SCFS is a powerful tool to determine the adhesion forces between viable cells and a surface of interest with pN force resolution [51]. This is to our knowledge the first time that this technology was used to investigate the initial adhesion behavior of the clinically important fungus *C. albicans* to clinically used CVC material in presence of infection-mimicking conditions. We could demonstrate that (i) germinated *C. albicans* cells adhere more efficiently to PU-based CVC surfaces than yeast cells, and (ii) that germinated cells adhere to HBP-coated surfaces under physiologically relevant flow rates in a flow directional orientation, in which most of the germ tubes exhibited angles of 150° to 210° deviating from the flow direction. Both findings suggest that the germ tube contributes significantly to the adhesion capacities of *C. albicans* to this clinically relevant implant material, and that germination processes and germ tube-associated adhesins such as Als3 play a bigger role in the initial adhesion of this fungal pathogen to implanted medical devices than currently assumed. This is supported by our finding that both specific virulence properties described above demonstrated to be almost extinguished when an *als3*Δ/*als3*Δ mutant strain was investigated. A comparative review by Soll and Daniels [52] highlights that variations in experimental conditions may have a tremendous effect on the biofilm composition and architecture. According to this review, any mechanical energy input by rotation, shaking, and flow, or alterations in nutrition are likely to exert an influence on the orientation and stratification of yeast cells and hyphae within the biofilm. Thus, different adherence mechanism to CVC (in the yeast phase or – like suggested in this work – promoted by germ tubes) might have an impact on the later formed biofilm structure on the CVC, which might possess new challenges or possibilities to treat catheter-related *C. albicans* infections.
